# Mapping of cholera hotspots in Kenya using epidemiologic and water, sanitation, and hygiene (WASH) indicators as part of Kenya’s new 2022–2030 cholera elimination plan

**DOI:** 10.1371/journal.pntd.0011166

**Published:** 2023-03-17

**Authors:** Catherine Kiama, Emmanuel Okunga, Annastacia Muange, Doris Marwanga, Daniel Langat, Francis Kuria, Patrick Amoth, Ian Were, John Gachohi, Nolluscus Ganda, Marion Martinez Valiente, M. Kariuki Njenga, Eric Osoro, Joan Brunkard

**Affiliations:** 1 Washington State University, Global Health Kenya, Nairobi, Kenya; 2 Kenya Ministry of Health, Nairobi, Kenya; 3 School of Public Health, Jomo Kenyatta University of Agriculture and Technology, Nairobi, Kenya; 4 World Health Organization, Nairobi, Kenya; 5 Global Task Force on Cholera Control, Geneva, Switzerland; 6 Paul G. Allen School for Global Health, Washington State University, Pullman, Washington, United States of America; 7 Centers for Disease Control and Prevention, Atlanta, Georgia, United States of America; Yale University School of Medicine, UNITED STATES

## Abstract

Cholera is an issue of major public health importance. It was first reported in Kenya in 1971, with the country experiencing outbreaks through the years, most recently in 2021. Factors associated with the outbreaks in Kenya include open defecation, population growth with inadequate expansion of safe drinking water and sanitation infrastructure, population movement from neighboring countries, crowded settings such as refugee camps coupled with massive displacement of persons, mass gathering events, and changes in rainfall patterns. The Ministry of Health, together with other ministries and partners, revised the national cholera control plan to a multisectoral cholera elimination plan that is aligned with the Global Roadmap for Ending Cholera. One of the key features in the revised plan is the identification of hotspots. The hotspot identification exercise followed guidance and tools provided by the Global Task Force on Cholera Control (GTFCC). Two epidemiological indicators were used to identify the sub-counties with the highest cholera burden: incidence per population and persistence. Additionally, two indicators were used to identify sub-counties with poor WASH coverage due to low proportions of households accessing improved water sources and improved sanitation facilities. The country reported over 25,000 cholera cases between 2015 and 2019. Of 290 sub-counties, 25 (8.6%) sub-counties were identified as a high epidemiological priority; 78 (26.9%) sub-counties were identified as high WASH priority; and 30 (10.3%) sub-counties were considered high priority based on a combination of epidemiological and WASH indicators. About 10% of the Kenyan population (4.89 million) is living in these 30-combination high-priority sub-counties. The novel method used to identify cholera hotspots in Kenya provides useful information to better target interventions in smaller geographical areas given resource constraints. Kenya plans to deploy oral cholera vaccines in addition to WASH interventions to the populations living in cholera hotspots as it targets cholera elimination by 2030.

## Author summary

Cholera is a major cause of diarrheal illness that can lead to severe dehydration and death within hours if left untreated. Kenya has experienced cholera outbreaks repeatedly in different parts of the country, including the refugee camps and informal settlements. To eliminate local transmission, a targeted approach to areas that are cholera prone is recommended. The findings of the hotspot mapping provide a better understanding of the smaller geographical areas in different parts of the country that have reported cholera outbreaks during the five years (2015–2019). The mapping also includes areas that have low access to improved water sources and improved sanitation facilities. The mapping findings documented here provide a rationale for prioritization of hotspots in the deployment of resource-intensive interventions, such as preventive vaccination campaigns and investment in water and sanitation infrastructure, that are key interventions to control cholera transmission.

## Introduction

Cholera is a leading cause of diarrheal illness globally with an estimated 1–4 million cases and tens of thousands of deaths occurring each year [1]. Most cases of the illness are mild, with severe illness in up to 20% of those infected. Death can occur within hours among severely dehydrated patients if treatment with oral rehydration or intravenous fluids is not received [2].

Cholera is an issue of major public health importance in Kenya and is one of the priority diseases under Kenya’s Integrated Disease Surveillance and Response (IDSR) strategy [3]. It was first reported in Kenya in 1971 in Turkana District and was associated with a pandemic originating in Southeast Asia in the 1960s [4]. The country has continued to report an upsurge of cases with large cyclical epidemics occurring approximately every five to seven years that last for two to three years. Notable widespread confirmed outbreaks affected various areas between 1997–1999, 2007–2010, and 2015 [5].

According to surveillance data from the Ministry of Health Kenya has experienced annual outbreaks with the highest number of cases recorded in 2015, declining in 2016, and steadily increasing from 2017 through to 2019. In the year 2020, a total of 711 cases, and 13 deaths were reported. In 2021, a total of 38 cases, and zero deaths were reported in the two refugee camps of Dadaab and Kakuma. In the first half of 2022, a total of 8 cases, and 2 deaths were reported in a cholera outbreak affecting the capital Nairobi.

Risk factors associated with outbreaks in Kenya include open defecation, population growth in slums and urban areas without adequate access to drinking water and sanitation infrastructure, cross-border movement of persons from neighboring countries experiencing complex humanitarian emergencies and large cholera outbreaks, crowded settings such as refugee camps coupled with massive displacement of persons, mass gathering events and changes in rainfall patterns [4].

In 2017, the World Health Organization-led Global Task Force on Cholera Control (GTFCC) launched a Global Roadmap for Ending Cholera to eliminate cholera transmission in up to 20 countries and reduce cholera mortality by 90% [6]. The Ending Cholera Roadmap was introduced at the World Health Assembly in 2018 and signed by more than 10 countries including Kenya [6]. As a follow-up to this commitment, Kenya revised its national multisectoral cholera elimination plan (NMCEP) to align with the Global Roadmap with the inclusion of hot spot mapping as a distinct new feature [7]. The implementation of the previous national plan followed a country-wide approach without specific targeting and prioritization of high disease-burden areas [8]. Here, we provide details on Kenya’s experience using the GTFCC tool to identify hotspots and its unique process of refining the hotspots by the inclusion of WASH data. Identifying targeted areas at risk for cholera will inform the prioritization of resource-intensive interventions such as the deployment of oral cholera vaccines (OCV) and water, sanitation, and hygiene (WASH) interventions, as Kenya implements its NMCEP in endemic sub-counties.

## Methods

### Study design

The analysis is based on retrospective data review with descriptive epidemiology of cholera burden and WASH coverage based on population census data.

### Study area

The identification of hotspots was conducted for the entire country. Kenya is administratively divided into 47 counties and further subdivided into 290 sub-counties [9]. A sub-county is defined as a decentralized unit through which the County governments of Kenya provide functions and services. Sub-counties were used as the unit of analysis for the identification of hotspots since they were the lowest subnational administrative unit where both case data and WASH data were available at the time of the analysis. The population of Kenya was 47.56 million according to the 2019 census and is projected to have increased to 53.49 million by 2022 [10].

### Study period

Data were assessed and analyzed from January 2015 to December 2019. This 5-year period included 260 epi weeks.

### Cholera surveillance and data sources

Cholera case data was extracted from the cholera case databases at the Division of Disease Surveillance and Response (DDSR) at the Ministry of Health, Kenya. The database comprises all cases reported to the DDSR including both suspected and confirmed cases. DDSR is responsible for detecting, assessment, notification, and reporting public health events including cholera to fulfill Kenya’s International Health Regulations (IHR) responsibilities.

Population data and WASH data by sub-county were sourced from the Kenya National Bureau of Statistics Census of 2019 [10].

### Standard case definitions

The standard case definitions used for cholera case reporting were guided by the IDSR Standard case definitions clinicians’ booklet [3].

○ A suspected case is defined as a patient age 5 years or more, presenting with acute, profuse, effortless watery diarrhea (3 or more times within 24 hours) with or without vomiting. If there is a cholera outbreak, a suspected case is any person aged 2 years and above with acute watery diarrhea, with or without vomiting.
○ A confirmed case is defined as a suspected case in which Vibrio cholera O1 or O139 has been isolated in the stool (confirmed by culture), or is epidemiologically linked to a confirmed case.

### Data cleaning and analysis

Following the deduplication of line list cholera surveillance case data, the aggregate number of cases and the number of weeks reporting cases were pooled by sub-county for each year. To identify the priority epidemiological cholera hotspots, the GTFCC’s Guidance and tool for countries to identify priority areas for intervention was utilized [11]. The guidance recommends the use of i) mean annual incidence and ii) persistence (both defined below) to quantify the historical incidence of cholera cases (both suspected and confirmed cases) and the persistence of cholera in an area. The tool utilizes Microsoft Excel and is available online for free [11]. Each row on the Microsoft Excel tool represents one sub-county. The following data were entered into the tool for each sub-county: a) population for each year, b) the number of reported cholera cases for each year, and c) the number of weeks reporting cases for each year. The GTFCC tool auto-calculated the following variables for each sub-county; a) annual incidence, b) mean annual incidence across the 5 years (January 2015 to December 2019), and c) proportion of weeks with reported cases.

In addition to the epidemiological indicators recommended by GTFCC, the Kenya analysis factored in two WASH indicators for each sub-county extracted from the census 2019 report: a) the proportion of households using an improved water source and b) the proportion of households using improved sanitation facilities. WASH indicators were defined according to the Joint Monitoring Program (JMP) criteria and the definitions are provided in the WASH indicators section below [12].

### Mean annual incidence (MAI) and persistence

Mean annual incidence (MAI) was calculated as an average of the annual incidence over 5 years [11]. Percentage persistence was calculated as the number of weeks in which cholera cases were reported out of the total 260 weeks expressed as a percentage [11].

**Fig 1:**
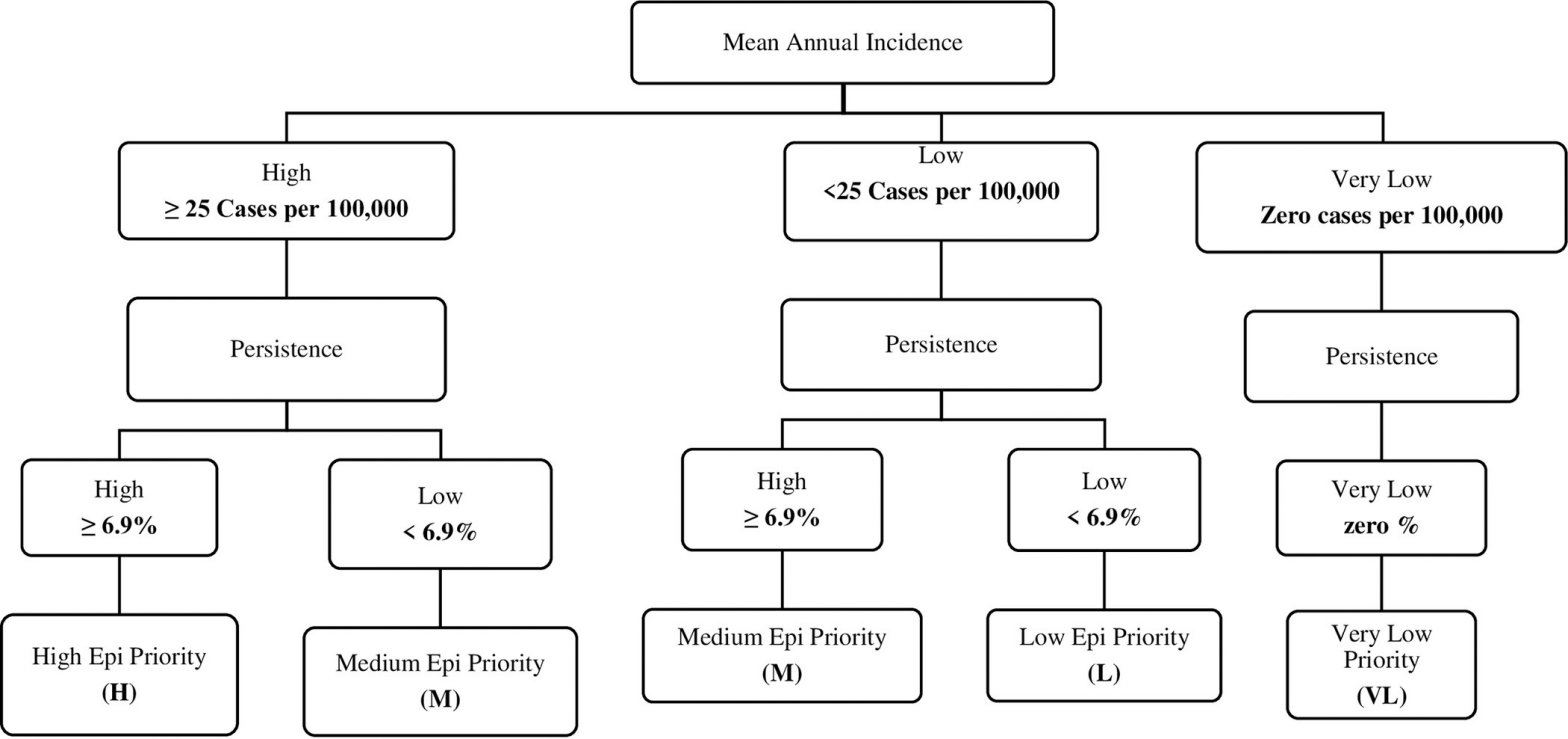
Prioritization by Epidemiological indicators.

Sub-counties with values above the national mean value were ranked as “High” and those below the mean were ranked as "Low” (Fig 1). The cut-off values were the mean national values for the two indicators i.e. 25 cases per 100,000 persons MAI, 6.9% weekly percentage persistence. Sub-counties were then categorized as high Epi priority (high incidence and high persistence of cholera), medium Epi priority (high incidence and low persistence or low incidence and high persistence of cholera), low Epi priority (low incidence and low persistence of cholera) or very low Epi priority (no cases reported).

### Water sanitation and hygiene (WASH) indicators

Improved drinking water sources are water sources that have the potential to deliver safe water by nature of their design and construction, and include piped water, boreholes or tube wells, protected dug wells, protected springs, rainwater, and packaged or delivered water [12]. The proportion of households with access to an improved water source is the percentage of households whose main source of drinking water was piped household water, a public tap or standpipe, tube-well or borehole, protected dug well, protected spring, collected rainwater or bottled water [12].

Improved sanitation is sanitation facilities designed to hygienically separate excreta from human contact, and include: flush/pour flush toilets connected to piped sewer systems, septic tanks or pit latrines; pit latrines with slabs (including ventilated pit latrines), and composting toilets [13]. Safely managed sanitation is the use of improved facilities that are not shared with other households and where excreta are safely disposed of in situ or removed and treated offsite The proportion of households with access to improved sanitation is the percentage of households with connection to the main sewers, septic tank, ventilated improved pit latrines or pit latrines with slabs, biodigesters or cesspools [13].

**Fig 2:**
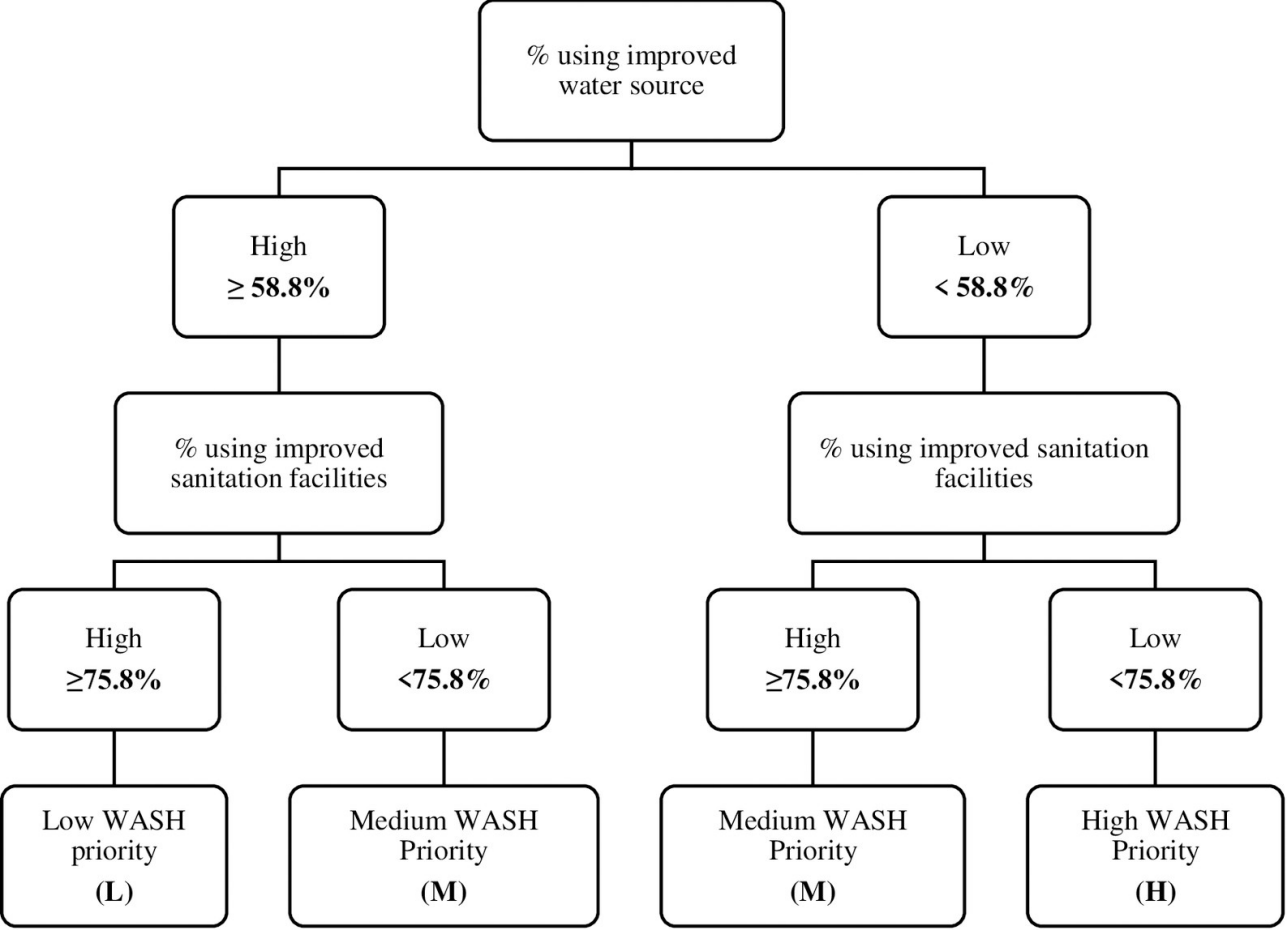
Prioritization by WASH indicators.

Sub-counties with values above the mean value were ranked as “High” and those below the mean were ranked as “Low” (Fig 2). The cut-off values were the mean national values for the two WASH indicators; 58.8% improved water sources, and 75.8% improved sanitation facilities. Sub-counties were then categorized as high WASH priority (low access to improved water sources and low access to improved sanitation facilities), medium WASH priority (low access to an improved water source and high access to improved sanitation facilities, or high access to an improved water source and low access to improved sanitation facilities) or low WASH priority (high access to an improved water source and high access to improved sanitation facilities).

### Epi, WASH, and combination priority categorizations

The WASH indicators were applied together with the 4 categories of epidemiological priority: high (H), medium (M), low (L), and very low (VL) epi priority to yield 12 possible outcomes (Table 1). The sub-counties were then scored from 12 to 1 (highest to lowest risk score). The risk scores were then translated into combination priority levels, i.e., high (score 9–12), medium (score 5–8), and low priority (score 1–4). Higher WASH priority sub-counties increased the risk categorization for the combination priority levels than the epi priority levels alone.

A cholera hotspot was defined as a "geographically limited area where environmental, cultural, and/or socioeconomic conditions facilitate the transmission of the disease and where cholera persists or re-appears regularly" [14].

**Table 1:** Priority categorization by 1. Epidemiological indicators, 2. WASH indicators and 3. a combination of both Epi and WASH.

Priority categorization	High (H)	Medium (M)	Low (L)	Very Low (VL)
1. Epi indicators	MAI ≥ 25 per 100,000 (H) & Pers ≥ 6.9%(H)	MAI ≥ 25 per 100,000 (H) & Pers < 6.9% (L) OR MAI < 25 per 100,000 (L) & Pers ≥ 6.9% (H)	MAI < 25 per 100,000 (L) & Pers < 6.9%(L)	MAI = zero per 100,000 (VL) & Pers = zero % (VL)
2. WASH indicators	Improved water < 58.8% (H) & Improved sanitation <75.8% (H)	Improved water < 58.8% (H) & Improved sanitation ≥ 75.8% (L) OR Improved water ≥ 58.8% (L) & Improved sanitation < 75.8% (H)	Improved water ≥ 58.8% (L) & Improved sanitation ≥ 75.8% (L)	N/A
3. Combination of Epi & WASH	Risk score 9–12 HH, HM, HL & MH	Risk score 5–8 MM, ML, LH & LM	Risk score 1–4 LL, VLH, VLM &VLL	N/A

High = H, Medium = M, Low = L, Very Low = VL, Not Applicable = N/A

### Validation of the methodologies and findings

Key stakeholders among them the Ministry of Health, Ministry of Water, county departments of health, and other partners participated in conceptualizing and implementing this study, including review and approval of the data sources and methodologies. The final report with findings of this exercise was subjected to validation and adopted by key stakeholders.

### Ethical considerations

Approval to access the line list data was sought from the Kenya Ministry of Health–Division of Disease Surveillance and Response. The study design did not require ethical approval as it used aggregated, de-identified data that had been collected for routine surveillance activities.

## Results

### Mean annual incidence and persistence

**Fig 3:**
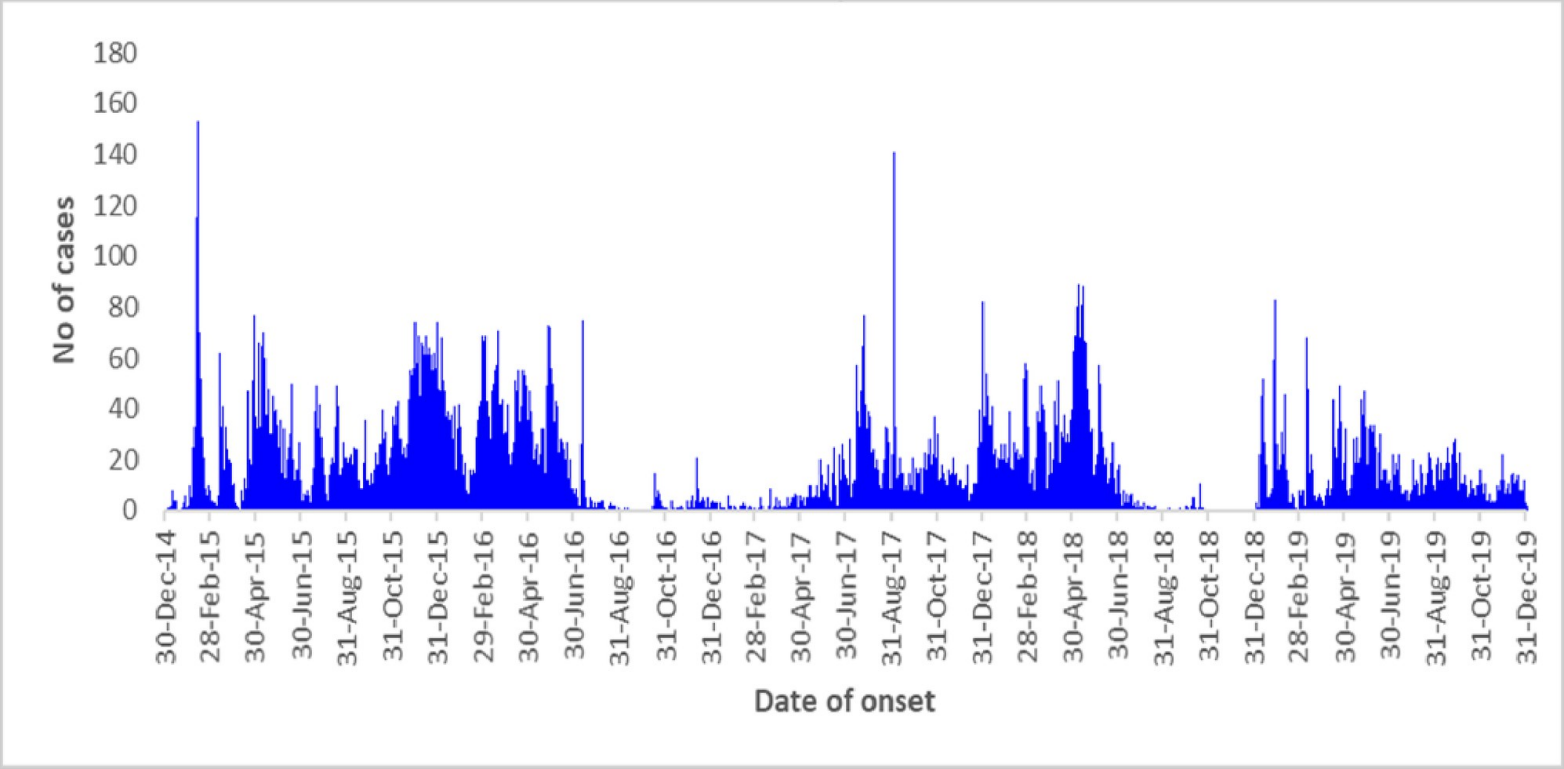
Epicurve showing the number of cholera cases reported by date of onset, Kenya, 2015–2019. Data source: Division of Disease Surveillance and Response, Ministry of Health, Kenya.

Cholera cases were reported in certain regions of the country throughout the 5 years (Fig 3). Cumulatively, a total of 25,992 cases and 479 deaths were reported from 2015–2019. The number of cases was highest (9815) in 2015 and lowest (2103) in 2016.

**Fig 4:**
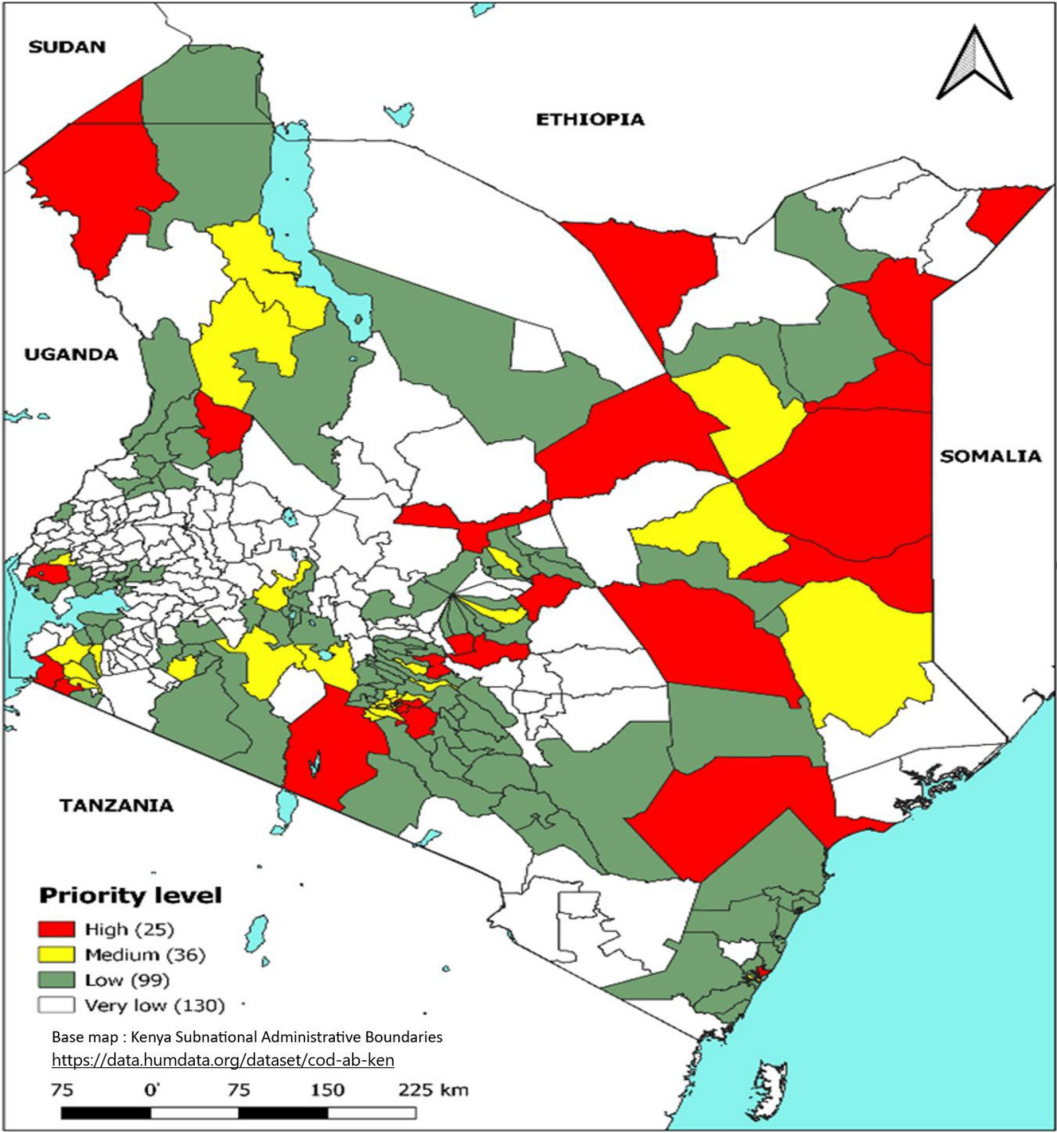
Priority sub-counties based on cholera MAI & Persistence, Kenya, 2015–2019. Link to base map layer: https://data.humdata.org/dataset/cod-ab-ken Link to terms of use: https://data.humdata.org/faqs/licenses.

A total of 130 sub-counties (44.8%) did not report any cholera cases during the period under review. Among the 160 sub-counties reporting cases, the mean number of cases was 180. The mean MAI was 25 cases per 100,000 population (range 0.09–531 cases per 100,000 population). The mean percentage persistence was 6.9% (range 0.38–56.5%). Using both MAI and persistence, 25 sub-counties (8.6%) were classified as a high priority, 36 (12.4%) as medium, 99 (34.1%) as low, and 130 (44.8%) did not report any cases as very low priority (Fig 4).

The 25 high Epi priority sub-counties accounted for 52.1% of the total cases. Sub-counties with the highest number of cases per population were in arid areas of Northern Kenya, those bordering other countries, and areas hosting refugee camps and urban informal settlements. Over the 5 years, refugee camps contributed 17.7% of the total cases: 14.3% from the larger Dadaab camp (population 233,805) primarily housing refugees from Somalia, and 3.4% from Kakuma camp (population 185,782) housing refugees primarily from South Sudan [15].

### WASH indicators

**Fig 5:**
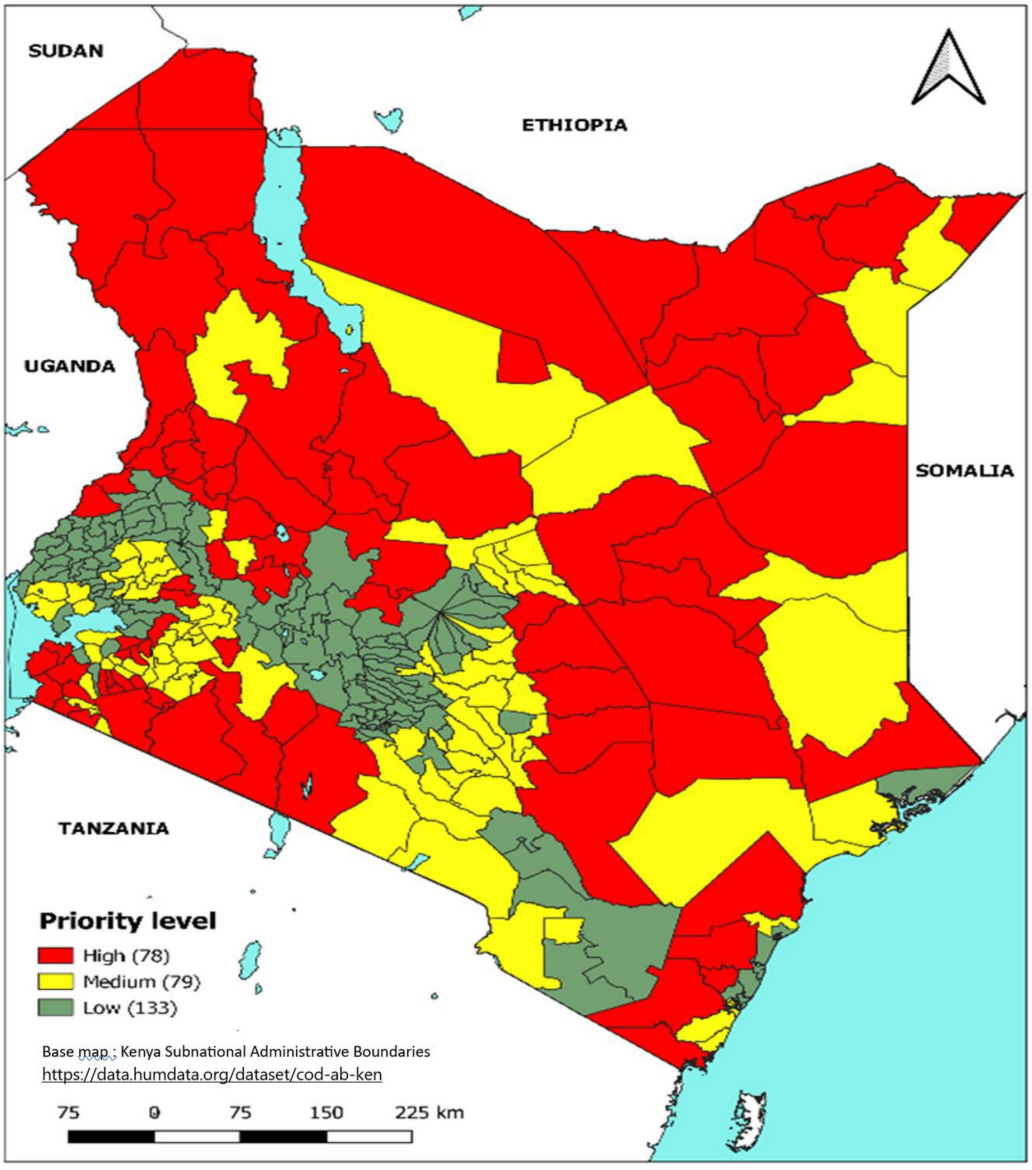
Priority sub-counties based on WASH indicators, Kenya, 2015–2019. Link to base map layer: https://data.humdata.org/dataset/cod-ab-ken Link to terms of use: https://data.humdata.org/faqs/licenses.

A total of 78 (26.9%) sub-counties were considered a high priority for WASH interventions based on improved water and improved sanitation access indicators (Fig 5). According to the 2019 census survey, the national average number of households with access to an improved water source, and households with access to improved sanitation facilities was 58.8% and 75.8% respectively.

### Combination of WASH and MAI/persistence indicators

**Fig 6:**
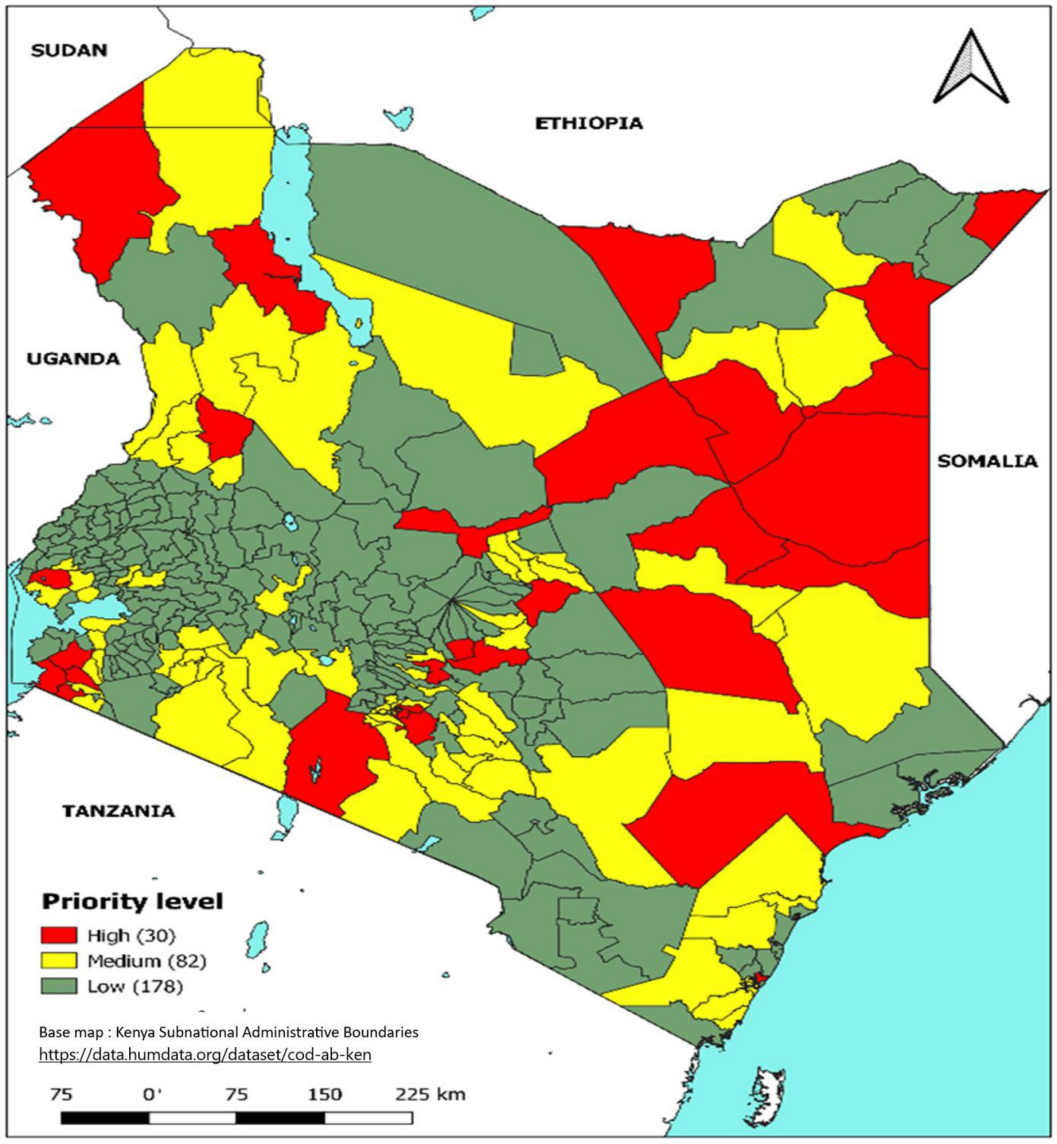
Priority sub-counties based on a combination of Epidemiological and WASH indicators, Kenya, 2015–2019. Link to base map layer: https://data.humdata.org/dataset/cod-ab-ken Link to terms of use: https://data.humdata.org/faqs/licenses.

**Fig 7:**
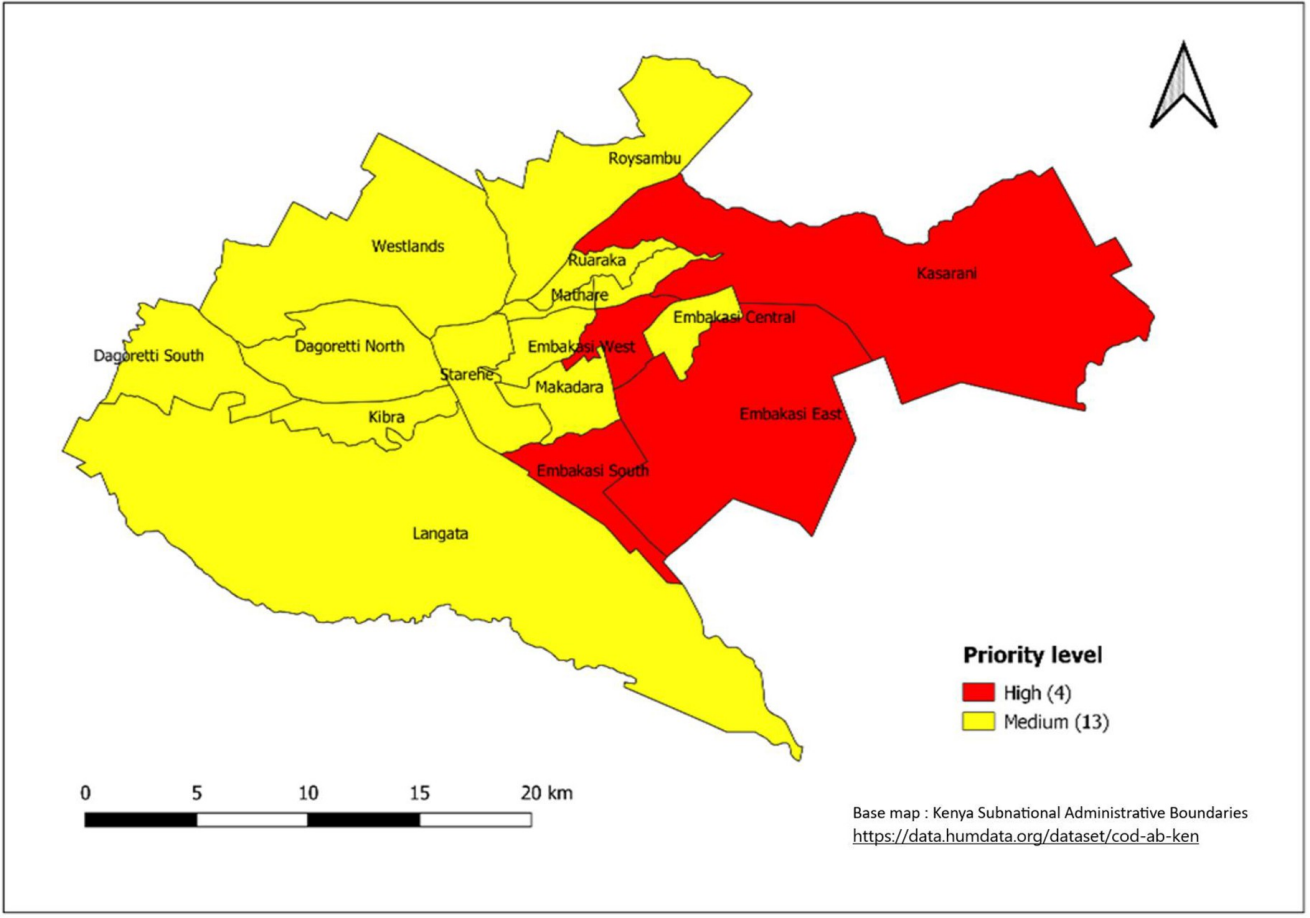
. Priority sub-counties in Nairobi County based on a combination of Epidemiological and WASH indicators, 2015–2019. Link to base map layer: https://data.humdata.org/dataset/cod-ab-ken Link to terms of use: https://data.humdata.org/faqs/licenses.

**Fig 8:**
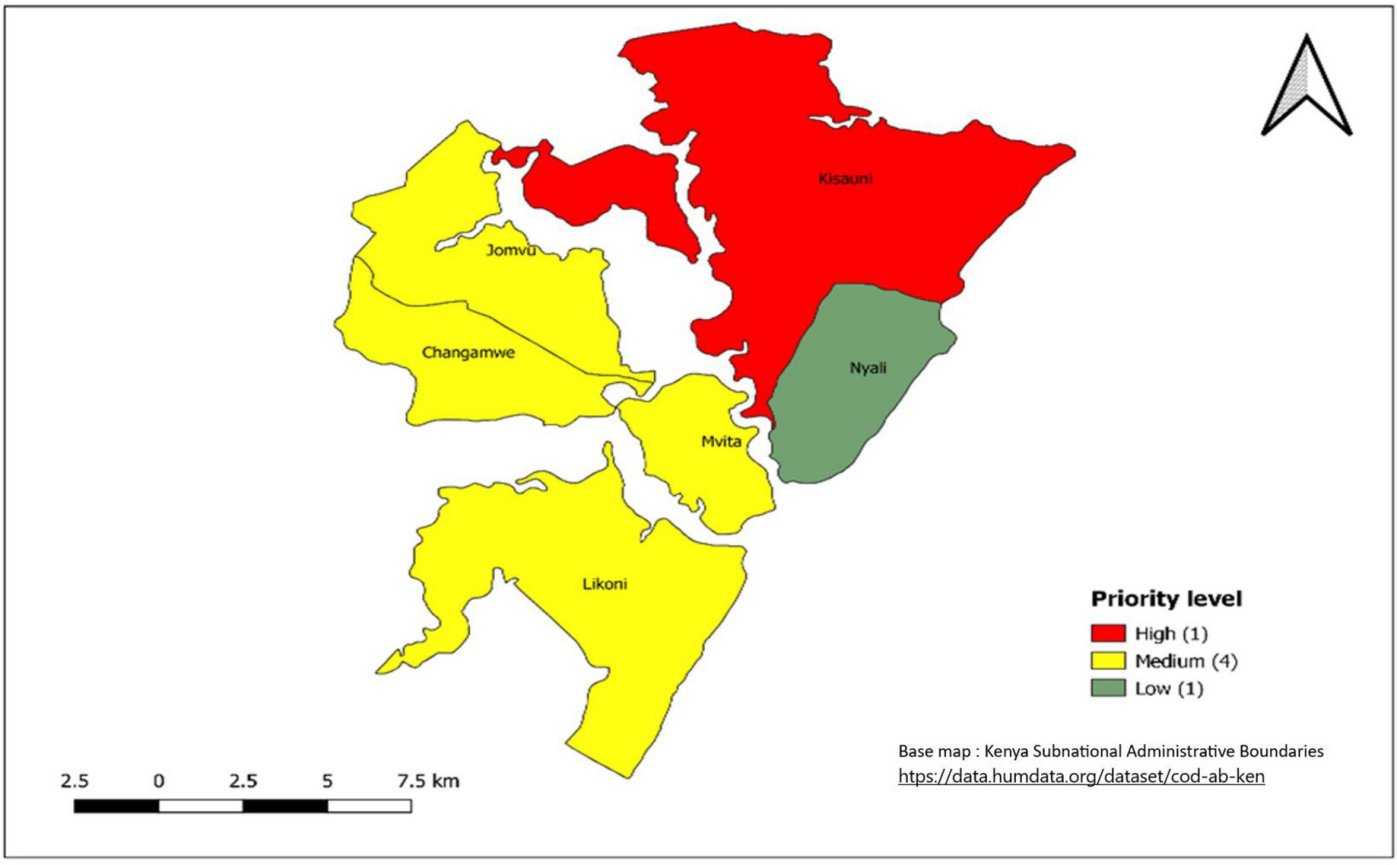
Priority sub-counties in Mombasa County based on a combination of Epidemiological and WASH indicators, 2015–2019. Link to base map layer: https://data.humdata.org/dataset/cod-ab-ken Link to terms of use: https://data.humdata.org/faqs/licenses.

**Fig 9:**
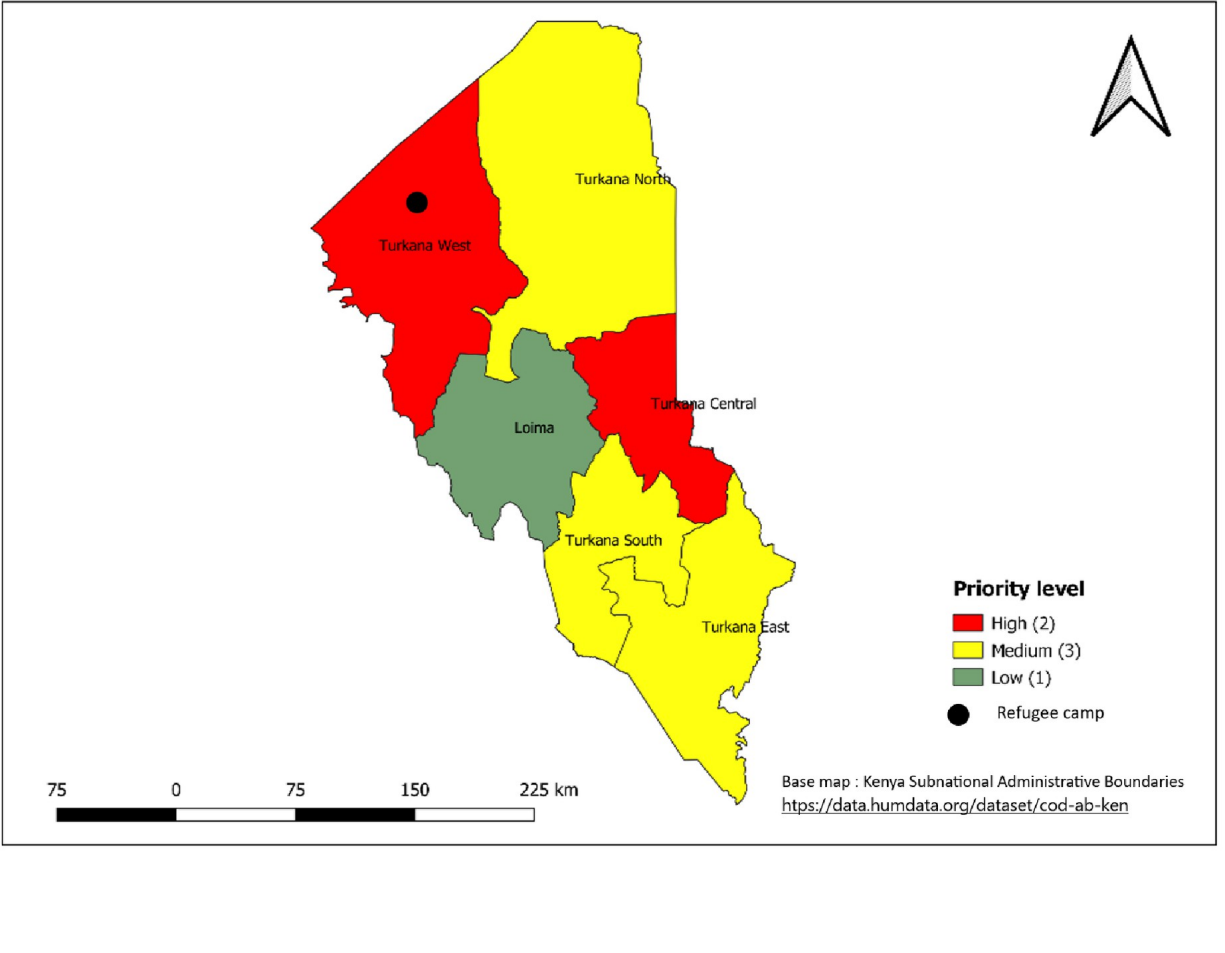
Priority sub-counties in Turkana County based on a combination of Epidemiological and WASH indicators, 2015–2019. Link to base map layer: https://data.humdata.org/dataset/cod-ab-ken Link to terms of use: https://data.humdata.org/faqs/licenses.

**Fig 10:**
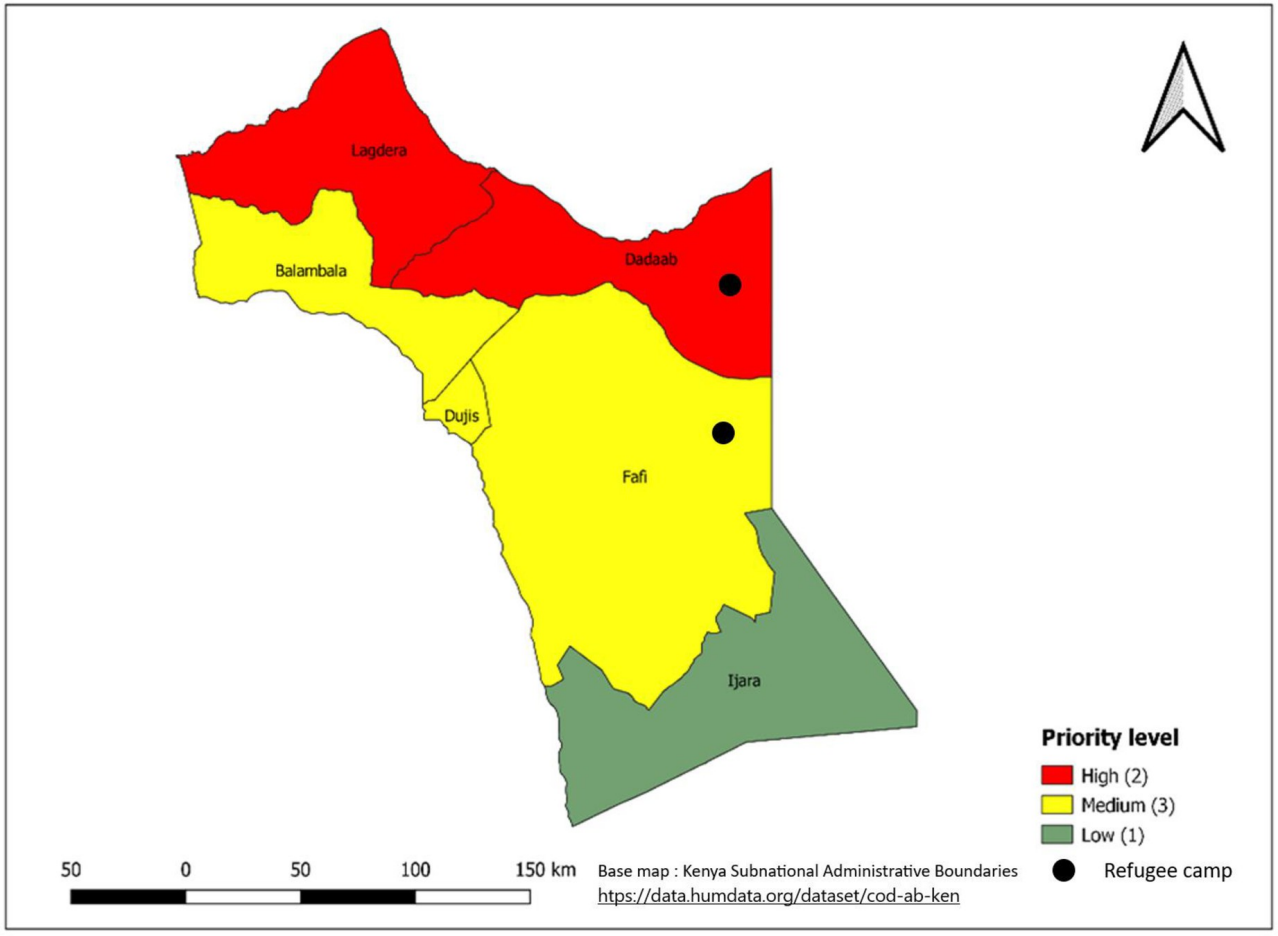
Priority sub-counties in Garissa County based on a combination of Epidemiological and WASH indicators, 2015- 2019. Link to base map layer: https://data.humdata.org/dataset/cod-ab-ken Link to terms of use: https://data.humdata.org/faqs/licenses.

After integrating the epidemiological and WASH indicators, 30 (10.3%) sub-counties were classified as a high priority, 82 (28.3%) as a medium priority and 178 (61.4%) as a low priority (Fig 6). The 30 high combination priority sub-counties are from the 19 counties of; Wajir, Turkana, Mandera, Tana River, Isiolo, Marsabit, Garissa, Kajiado, Homabay, Migori, Tharaka Nithi, Siaya, West Pokot, Kirinyaga, Embu, Mombasa, Murang’a, Machakos and Nairobi (Table 2, Figs 6–10).

**Table 2:** List of High priority sub-counties after a combination of epidemiological and WASH indicators in Kenya, 2015–2019.

Priority level after a combination of Epi &WASH indicators	Number of sub-counties	Name of County (sub-counties)
High	30	**Wajir** (Wajir South, Wajir East, Wajir West) **Turkana** (Turkana West, Turkana Central) **Mandera** (Mandera South, Mandera East) **Tana River** (Bura, Garsen) **Isiolo** (Isiolo North) **Marsabit** (Moyale) **Garissa** (Daadab, Lagdera) **Kajiado** (Kajiado West) **Homabay** (Ndhiwa) **Migori** (Nyatike, Suna West, Uriri) **Tharaka Nithi** (Tharaka) **Siaya** (Alego Usonga) **West Pokot** (Sigor) **Kirinyaga** (Mwea) **Embu** (Gachoka) **Mombasa** (Kisauni) **Murang’a** (Maragwa) **Machakos** (Mavoko) **Nairobi** (Embakasi West, Embakasi South, Embakasi East, Kasarani)

The population living in the 30-combination high-priority sub-counties was 9.1% (4,892,015) of the total population in Kenya while that in medium-priority sub-counties was 32.8% (17,634,859) (Table 3).

**Table 3:** Population sizes by priority level.

**Priority level after a combination of Epi &WASH indicators**	**Risk score**	Combination of Epi & WASH indicators*	**No. of Sub-counties**	**Population size**	**Cumulative population**	**Proportion (%) of the total Population**
High	12	HH	10	1,513,535	1,513,535	2.8
	11	HM	8	1,236,414	2,749,949	5.1
	10	HL	7	1,394,275	4,144,224	7.7
	9	MH	5	747,790	4,892,015	9.1
Medium	8	MM	8	1,392,213	6,284,228	11.7
	7	ML	23	7,252,298	13,536,526	25.2
	6	LH	19	2,677,740	16,214,266	30.2
	5	LM	32	6,312,608	22,526,874	41.9
Low	4	LL	48	9,838,639	32,365,513	60.2
	3	VLH	44	6,310,940	38,676,453	72.0
	2	VLM	31	5,228,360	43,904,813	81.7
	1	VLL	55	9,845,426	53,750,240	100.0

*High = H, Medium = M, Low = L, Very Low = VL

## Discussion

Since the first case of cholera was reported in Turkana in 1971, Kenya has consistently reported outbreaks, except for a few years. In this hotspot identification exercise, cholera was reported each consecutive year from 2015 through 2019 (Fig 3). We found that the disease did not occur uniformly across the country but in smaller geographical areas. The majority of sub-counties that did not report any cases over the 5 years were from the central part of the country where the climate is temperate (Fig 4). A few of the sub-counties that did not report cases were from the arid and semi-arid zones including those adjacent to sub-counties with high incidence and persistence.

The 30 sub-counties that were mapped as high priority account for more than half of the cholera cases reported during 2015–2019. The high-priority sub-counties (Table 2, Fig 4) were those in the arid and semiarid zones of the country towards the north of Kenya, bordering neighboring countries that experience cholera outbreaks or conflict (i.e., Somalia, Ethiopia, Tanzania, and South Sudan), urban informal settlements of Nairobi and Mombasa (Figs 7 and 8), areas hosting large refugee camps (Figs 9 and 10), areas with communities that are predominantly pastoralist, transport corridors transiting to other countries, and areas around large water bodies such as Lake Turkana, Lake Victoria and irrigation schemes in Mwea. Likewise, in neighboring South Sudan, refugees, informal settlements with displaced persons, nomadic pastoralists, major commercial hubs, major cities bordering other countries, and communities along major rivers were mapped as high-priority populations [16,17]. International borders are high-priority areas in most African countries suggesting cross-border disease spread [17,18].

Notably, over 50% of the country is mapped as either high or medium WASH priority (Fig 5). Similarly, a survey in Ethiopia reported the proportion of households using unimproved water sources is high and far from the UN Sustainable Development Goal (SDG) or the national goal [19,20]. As Kenya implements interventions toward elimination, the country must invest in the expansion of WASH infrastructure to increase access to improved water and sanitation. The application of WASH indicators to the recommended epidemiological indicators helped to identify areas that are most vulnerable to cholera due to the poor WASH conditions yet where cholera incidence and persistence were reported as either medium, low, or very low epi priority possibly due to incomplete reporting in these areas. Sub-counties with high WASH priority were elevated in the risk scoring given the heightened risk for disease transmission associated with inadequate WASH access; these were primarily located in the arid and semiarid zones to the north of Kenya, areas along the Kenyan coast, the western part of the country and in the central part of Kenya. A total of 5 sub-counties from the arid and semi-arid areas were elevated from medium Epi to high priority after combination (category MH) due to poor WASH conditions (Table 3).

Approximately 10% of the population is living in high-priority hotspots in Kenya and is vulnerable to the disease (Table 3). The risk scoring that combines both epidemiological and WASH indicators allows for stepwise inclusion of other populations at risk should resources for OCV or other interventions become available. In South Sudan, almost 20% of its population was mapped as vulnerable and approximately 2 million OCV doses were administered between 2012 and 2017 guided by the country’s hotspot mapping [16,17]. Other countries that have recently used hotspot mapping to guide the targeting of interventions include Uganda, Zambia, Burundi, and Ethiopia [18,20–22]. Nigeria has also recently used the GTFCC method to map its’ high-priority areas [23]. Mapping of the cholera burden using incidence data from sub-Saharan Africa found that although the disease was reported throughout the region, the highest incidence areas comprise a small proportion (4%) of the continent [24]. By deploying effective targeted interventions, the study found that prioritizing high-risk areas could substantially increase the efficiency of cholera control programs and could eliminate 50% of the region’s cholera cases by covering 4% population with vaccination [24].

This analysis is subject to several limitations. First, the analysis assumes that all sub-counties had complete and uniform reporting throughout the 5 years and that a zero denotes no cases as opposed to a lack of a report, resulting in the possibility that cases were underreported in some sub-counties. Further, the analysis assumes that all cases reported from a sub-county were resident in the same area and not from the neighboring sub-counties or in transit. Additionally, the WASH coverage data applied in this analysis does not equate to the coverage of safe water free from contaminants or safely managed sanitation which is most likely lower than the proportions provided.

## Conclusion

The identification of cholera hotspot sub-counties provides valuable information to better target prevention efforts in smaller geographical areas, especially resource-intensive interventions such as the use of OCV and WASH infrastructure expansion. The results further demonstrate the need for multisectoral collaboration toward cholera elimination. Almost half of the country has poor access to improved water sources and sanitation which falls within the mandate of other ministries and actors beyond the Ministry of Health. As part of the multisectoral approach, the information generated will be shared with multiple sectors and relevant stakeholders to form a basis for prioritization.

The GTFCC tool provides a simple and standardized approach that is easy to use and available online at no cost. The tool allows for analysis at various administrative levels from national to subnational and even smaller administrative units where data is available. The tool also allows for the addition of more indicators where data is available such as the application of select WASH indicators, which were instrumental in Kenya’s hotspot prioritization process.

## Ethics statement

Ethical review and approval were waived for this analysis based on retrospective data.

## Supporting information

S1 Table Summary table for identification of hotspots. H = High, M = Medium, L = Low, VL = Very Low.
(XLSX)

## References

[1] Cholera [Internet]. [cited 2022 Jun 13]. Available from: https://www.who.int/health-topics/cholera.

[2] Cholera [Internet]. [cited 2022 Jun 13]. Available from: https://www.who.int/news-room/fact-sheets/detail/cholera.

[3] Standard_Case_Definitions_for_Priority_Diseases_in_Kenya-_Integ.pdf [Internet]. [cited 2022 Jun 13]. Available from: http://guidelines.health.go.ke:8000/media/Standard_Case_Definitions_for_Priority_Diseases_in_Kenya-_Integ.pdf.

[4] Cowman G, Otipo S, Njeru I, Achia T, Thirumurthy H, Bartram J, et al. Factors associated with cholera in Kenya, 2008–2013. Pan Afr Med J. 2017 Oct 3;28:101. doi: 10.11604/pamj.2017.28.101.12806 29515719 PMC5837167

[5] Cholera–Kenya [Internet]. [cited 2022 Jun 13]. Available from: https://www.who.int/emergencies/disease-outbreak-news/item/21-july-2017-cholera-kenya-en.

[6] gtfcc-ending-cholera-a-global-roadmap-to-2030.pdf [Internet]. [cited 2022 Jun 13]. Available from: https://www.gtfcc.org/wp-content/uploads/2019/10/gtfcc-ending-cholera-a-global-roadmap-to-2030.pdf.

[7] Development of Kenya NCP–Global Task Force on Cholera Control [Internet]. [cited 2022 Jun 13]. Available from: https://www.gtfcc.org/news/development-of-kenya-ncp/.

[8] cholera_plan-final_21_11.pdf [Internet]. [cited 2022 Jun 13]. Available from: https://www.humanitarianresponse.info/sites/www.humanitarianresponse.info/files/documents/files/cholera_plan-final_21_11.pdf.

[9] IEBC Boundaries [Internet]. [cited 2022 Jun 13]. Available from: https://www.iebc.or.ke/uploads/resources/oep20PEuYn.pdf.

[10] adminusr. 2019 Kenya Population and Housing Census Results [Internet]. Kenya National Bureau of Statistics. 2019 [cited 2022 Jun 13]. Available from: https://www.knbs.or.ke/2019-kenya-population-and-housing-census-results/.

[11] guidance-and-tool-for-countries-to-identify-priority-areas-for-intervention.pdf [Internet]. [cited 2022 Jun 13]. Available from: https://www.gtfcc.org/wp-content/uploads/2019/11/guidance-and-tool-for-countries-to-identify-priority-areas-for-intervention.pdf.

[12] Drinking water | JMP [Internet]. [cited 2022 Jun 13]. Available from: https://washdata.org/monitoring/drinking-water.

[13] Sanitation | JMP [Internet]. [cited 2022 Jun 13]. Available from: https://washdata.org/monitoring/sanitation.

[14] gtfcc-interim-guidance-document-on-cholera-surveillance.pdf [Internet]. [cited 2022 Jun 13]. Available from: https://www.gtfcc.org/wp-content/uploads/2019/10/gtfcc-interim-guidance-document-on-cholera-surveillance.pdf.

[15] Kenya-Statistics-Package-30-June-2022.pdf [Internet]. [cited 2022 Jul 28]. Available from: https://www.unhcr.org/ke/wp-content/uploads/sites/2/2022/07/Kenya-Statistics-Package-30-June-2022.pdf.

[16] Cholera epidemiology in South Sudan_UNICEF_April 2018_FINAL.pdf [Internet]. [cited 2022 Jun 13]. Available from: https://plateformecholera.info/attachments/article/639/Cholera%20epidemiology%20in%20South%20Sudan_UNICEF_April%202018_FINAL.pdf.

[17] Vinvyi JFW. cholera situation analysis and hotspot mapping in south Sudan.: 19.

[18] Bwire G, Ali M, Sack D, Nakinsige A, Naigaga M, Debes A, et al. Identifying cholera “hotspots” in Uganda: An analysis of cholera surveillance data from 2011 to 2016. PLoS Negl Trop Dis. 2017 Dec 28;11:e0006118. doi: 10.1371/journal.pntd.0006118 29284003 PMC5746206

[19] Bogale GG. Hotspots of unimproved sources of drinking water in Ethiopia: mapping and spatial analysis of Ethiopia demographic and health survey Data 2016. BMC Public Health. 2020 Jun 8;20(1):878. doi: 10.1186/s12889-020-08957-2 32513128 PMC7278129

[20] Tesfay N, Biru M. Three Consecutive Waves of Cholera Outbreak… Three Consecutive Waves of Cholera Outbreak in Ethiopia (2015–2017): Explanatory Analysis. Ethiop J Health Sci. 2015 Jan 1;30:469.

[21] Mwaba J, Debes AK, Shea P, Mukonka V, Chewe O, Chisenga C, et al. Identification of cholera hotspots in Zambia: A spatiotemporal analysis of cholera data from 2008 to 2017. PLoS Negl Trop Dis. 2020 Apr;14(4):e0008227. doi: 10.1371/journal.pntd.0008227 32294084 PMC7159183

[22] Debes AK, Shaffer AM, Ndikumana T, Liesse I, Ribaira E, Djumo C, et al. Cholera Hot-Spots and Contextual Factors in Burundi, Planning for Elimination. Trop Med Infect Dis. 2021 May 11;6(2):76. doi: 10.3390/tropicalmed6020076 34064986 PMC8163194

[23] Ngwa MC, Ihekweazu C, Okwor TJ, Yennan S, Williams N, Elimian K, et al. The micro-hotspots of cholera in Kano State, Nigeria, 2010–2019—analysis of patient characteristics, Spatio-temporal patterns, and contextual determinants at the ward level [Internet]. medRxiv; 2021 [cited 2022 Jun 13]. p. 2021.08.20.21262313. Available from: https://www.medrxiv.org/content/10.1101/2021.08.20.21262313v1

[24] Mapping the burden of cholera in sub-Saharan Africa and implications for control: an analysis of data across geographical scales.—Abstract—Europe PMC [Internet]. [cited 2022 Jun 13]. Available from: https://europepmc.org/article/pmc/pmc5946088. 10.1016/S0140-6736(17)33050-7 PMC5946088 29502905

